# A Quality Assessment Network for Failure Detection in 3D Printing for Future Space-Based Manufacturing

**DOI:** 10.3390/s23104689

**Published:** 2023-05-12

**Authors:** Jianning Tang, Xiaofeng Wu

**Affiliations:** School of Aerospace, Mechanical and Mechatronic Engineering, The University of Sydney, Sydney 2006, Australia; jtan7551@uni.sydney.edu.au

**Keywords:** 3D printing, space manufacturing, quality assessment, machine learning

## Abstract

The application of space manufacturing technology holds tremendous potential for the advancement of space exploration. With significant investment from respected research institutions such as NASA, ESA, and CAST, along with private companies such as Made In Space, OHB System, Incus, and Lithoz, this sector has recently experienced a notable surge in development. Among the available manufacturing technologies, 3D printing has been successfully tested in the microgravity environment onboard the International Space Station (ISS), emerging as a versatile and promising solution for the future of space manufacturing. In this paper, an automated Quality Assessment (QA) approach for space-based 3D printing is proposed, aiming to enable the autonomous evaluation on the 3D printed results, thus freeing the system from reliance on human intervention, an essential requirement for the operation of space-based manufacturing platforms functioning in the exposed space environment. Specifically, this study investigates three types of common 3D printing failures, namely, indentation, protrusion, and layering to design an effective and efficient fault detection network that outperforms its counterparts backboned with other existing networks. The proposed approach has achieved a detection rate of up to 82.7% with an average confidence of 91.6% by training with the artificial samples, demonstrating promising results for the future implementation of 3D printing in space manufacturing.

## 1. Introduction

In recent years, space manufacturing has become a focus of interest due to several factors, including the high cost of launching payloads into space [1], limitations on the dimensions of payloads that can be transported on space shuttles [2], the need to support long duration space missions [3], and the prospect of future space colonization [4]. Three dimensional printing technology has significant potential for space applications due to its unique advantages. The ability to manufacture on-demand and in situ during long duration space flights can substantially reduce the mass required for maintenance and resupply purposes as more supplies can be produced on-orbit using raw materials [5,6]. Numerous institutes and private companies are currently exploring 3D printing technology for space applications. NASA, in collaboration with its commercial partner Made In Space, Inc., has developed a 3D printer that can operate onboard the International Space Station (ISS) and successfully printed samples in microgravity environments [7]. The samples have been tested and compared to those produced on the ground, demonstrating that microgravity has no significant impact on the samples [8]. This result confirms the feasibility of applying Fused Filament Fabrication (FFF) technology in space and sets the stage for its future development. The newly developed 3D printer onboard the ISS has since produced various functional parts, including antenna parts, probe adaptors, and connectors [3]. Currently, ESA is also engaged in 3D printing research for space applications. Through collaborations with its commercial partners, ESA aims to manufacture facilities on the moon to support future missions. In another joint project, ESA is partnering with Incus, OHB System AG, and Lithoz GmbH to develop a 3D printing technology that can use metal powder recycled from scrap metal, which can reuse the scraped metals in space and further reduce the logistic and delivery cost [9]. Meanwhile, China Academy of Space Technology (CAST) has successfully tested the its 3D printing technology on-orbit in a new unmanned spacecraft, which means the entire printing process is less supervised by human than the testing performed on ISS. Particularly, the CAST uses continuous carbon fiber-reinforced polymer composites as the printing material which demonstrates better electrical and mechanical performances than conventional polymers used in 3D printing material (PLA and ABS) [10]. Besides the hardware planned to be additively manufactured for space missions, the space-based 3D printing can be applied in multiple areas to support future space exploration. The crew health is critical for the mission success, however, maintaining adequate nutrition supply to astronauts is becoming harder in further deep space missions. BeeHex as a private commercial company is developing a new 3D printer for space use to provide healthy food to the astronauts in space. Currently, a 3D printer named Chef 3D developed by BeeHex is capable of printing decorations on cakes and cookies [11].

The application of 3D printing in space manufacturing offers notable advantages, such as the ability to fabricate complex objects without the need for additional assembly and the flexibility in the design-to-manufacturing process, resulting in less waste of raw materials. Additionally, various materials including metals, polymers, ceramics, and concrete, can be used in 3D printing. In addition to the forementioned FFF technique, various techniques such as selective laser sintering (SLS), stereolithography (SLA), and direct energy deposition (DED) are commonly used for 3D printing [12]. In conventional cases, the operator must monitor the printing process and check the products for defects. However, in the case of space manufacturing, the printing system must operate with minimal human supervision, and the quality of printed parts is critical as defects could lead to failures during operations. Therefore, in space 3D printing, where human presence is limited or not feasible, automated quality assessment (QA) methods are crucial to ensure reliable and efficient printing operations. Deep convolutional neural networks (CNNs) offer the potential to enhance the automatic operation of space-based printing systems by providing an intelligent and automated QA method that can detect printing failures, assess print quality, and make decisions without human intervention. This advancement is particularly crucial in the context of space 3D printing, where human presence is limited, and real-time decision-making is essential for efficient and reliable printing operations. Despite a highly automated printing system with integrated quality assessment function is required, it is yet to be developed. Therefore, research in this area is crucial for the successful implementation of space-based 3D printing.

Computer vision could be a feasible method to assess the 3D printed parts and detect failures. The complex failure modes of 3D printed parts, such as denting, protrusion, layering, and cracking, make it challenging to use traditional camera-based detection methods that rely on manually selected features and fixed detection rules, as the failures are changeable in both size and shape. However, computer vision methods using deep convolutional neural networks (CNNs) offer a promising solution due to their demonstrated ability to learn robust discriminative features from images and handle large variations. CNNs have become a core technology in the field of image processing [13] and have achieved state-of-the-art results in various computer vision tasks [14]. Therefore, using CNNs to automatically assess the quality of 3D printed parts is a feasible approach. Additionally, YOLOv3 has been widely used in industry because of its advantages on computation resources saving, board compatibility and faster FPS than YOLOv4 and YOLOv5 [15,16]. In this paper, the YOLOv3 detection head is used to detect the proposed failures on the 3D printed pieces. Compared to the counterparts designed for conventional use, the varying brightness or lighting conditions in space environment is an additional factor, which can impact failure detection results. The research in this area is still inadequate. Training a CNN-based QA method on samples with varying brightness can improve its performance in detecting a range of printing failure modes, such as printing failure, surface defects, or structural deformations, under different lighting conditions. Additionally, the proposed CNN-based network architecture is designed to be compact in size, which can be advantageous for space-based printing systems where size and weight constraints are critical factors.

Three dimensional printing in space can face challenges such as printing failures or damage during operation, which may result in material waste and impact mission efficiency. The proposed QA method holds promise for supporting on-orbit repair of 3D printed parts by providing real-time and automated assessment of print quality. By identifying and localizing printing failures or defects, the QA method can guide the repair process, such as patching or re-printing damaged areas, without the need for replacement or returning the parts to Earth. This can potentially minimize material waste arising from printing failures and damage during operation, leading to more sustainable and cost-effective space missions.

The contributions of this paper include:

1. The proposed deep convolutional neural network (CNN)-based quality assessment (QA) method for 3D printing has the potential to enhance the automatic operation of space-based printing systems with minimal human intervention. This advancement is particularly crucial in the context of space 3D printing, where human presence is limited.

2. The proposed CNN-based network architecture trained on sample with varying brightness exhibits superior performance in detecting a range of 3D printing failure modes, while also featuring a compact size in comparison to other variants.

3. The proposed QA method also holds promise for supporting on-orbit repair of 3D-printed parts, which could minimize material waste arising from printing failures and damage during operation.

## 2. Mission Description

**Figure 1:**
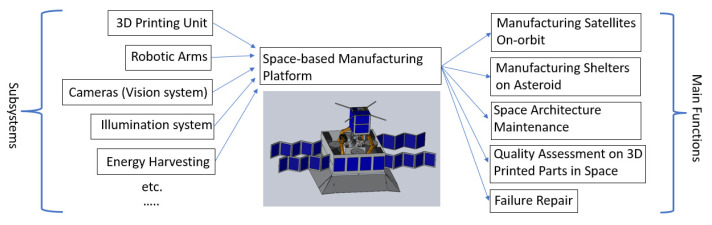
The concept of the on-orbit manufacturing system.

This study is a constituent part of the flagship project “On-orbit Manufacturing Platform” in the USYD (University of Sydney) Space Lab. The overall concept is depicted in Figure 1. The project aims to develop a 3D printing platform capable of automated operation in exposed space environment for performing various manufacturing tasks. These tasks include producing spare parts to service satellites in orbit and constructing tools and shelters on planets to facilitate human long duration missions in space. The platform comprises several modules, such as the printing structure, robotic arms, and vision camera system, etc. While previous studies have explored the utilization of robotic arms and 3D printing systems in space environments [17,18], this study centers on the development of a Quality Assessment (QA) system with fault tolerance ability based on the vision cameras integrated with the platform.

## 3. Methods

### 3.1. Architecture of the Proposed Neural Network

The availability of limited memory and computational resources onboard spacecraft presents a bottleneck for running big neural networks in the context of on-orbit 3D printing. This limitation constrains the complexity of the network that can be deployed. As such, an eligible neural network architecture for this purpose should be compact in size without trading off its performance.

The proposed network in this study is based on Inception-ResNet-V2 [14], which has been improved by optimizing input resolution [19,20]. Inception-ResNet-V2 was utilized as the foundational structure due to its notable advantages. The combination of inception modules and residual connections enables more efficient feature extraction at different scales and improved gradient flow during training, resulting in higher accuracy. Specifically, the network achieved 17.8% Top-1 Error and 3.7% Top-5 Error on the full set of 50 thousand images in the ILSVRC 2012 dataset [21]. Moreover, the computational cost of Inception-ResNet-V2 is lower than that of traditional networks, which is a crucial factor for the present study since limited computation resources are available on the satellite. Inception-ResNet-V2 architecture includes inception modules that are computationally efficient, utilizing different kernel sizes in a parallel structure, thereby enabling the network to capture features more efficiently with fewer parameters than conventional architectures. Additionally, the combination of residual connections and inception modules enhances the robustness of the Inception-ResNet-V2 architecture to image variations such as scale, direction, and resolution [22,23].

**Figure 2:**
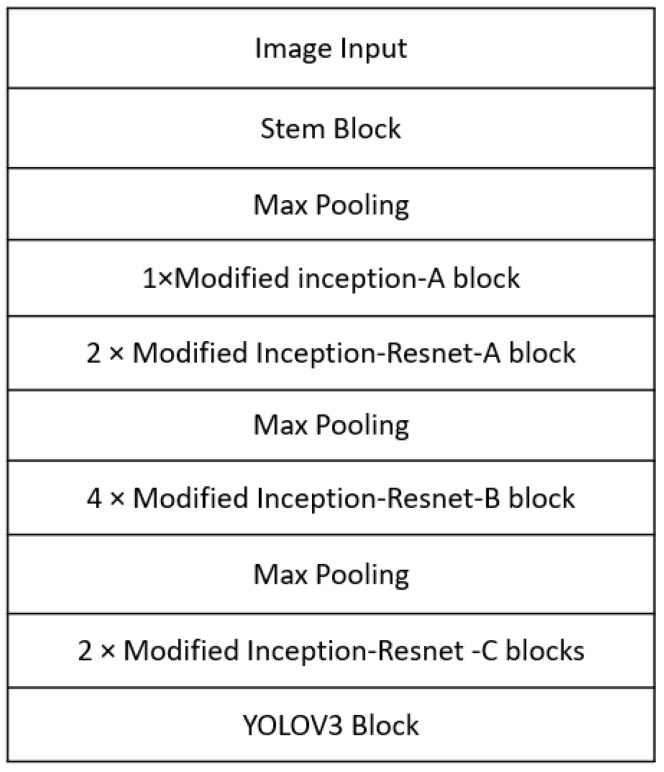
The schema of the 3D-QAnet used for 3D printing failure detection in space.

**Figure 3:**
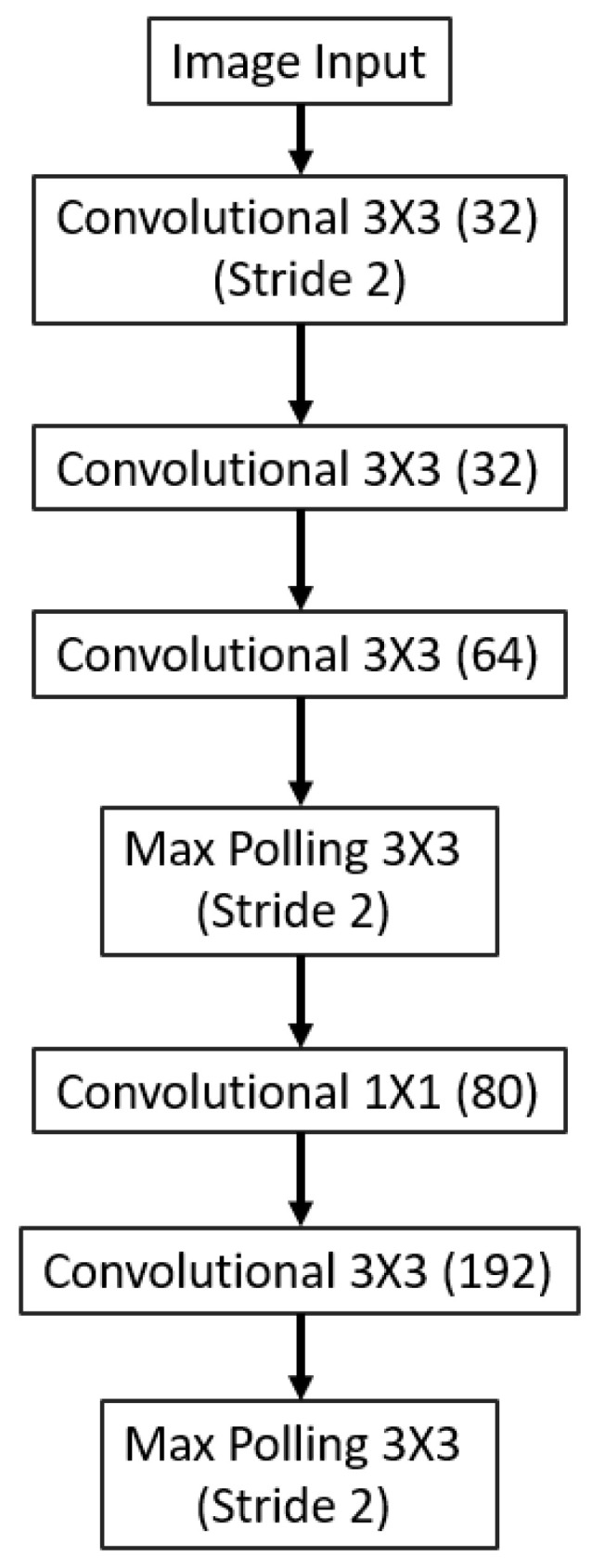
Stem block with image input layer.

**Figure 4:**
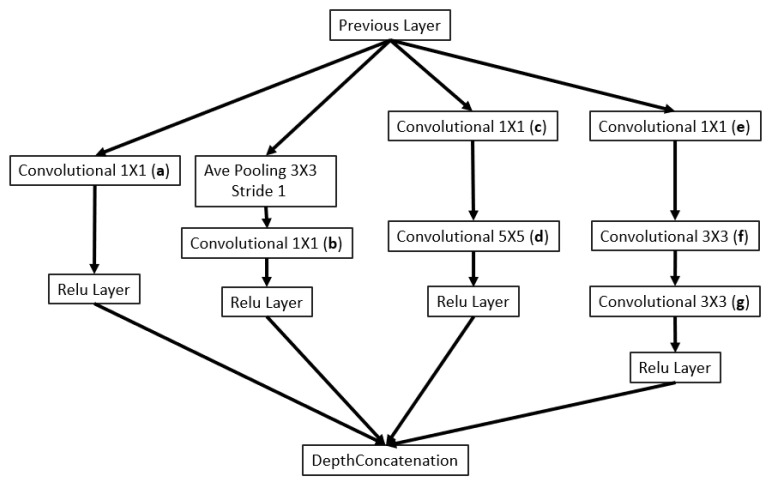
Modified Inception-A block.

**Figure 5:**
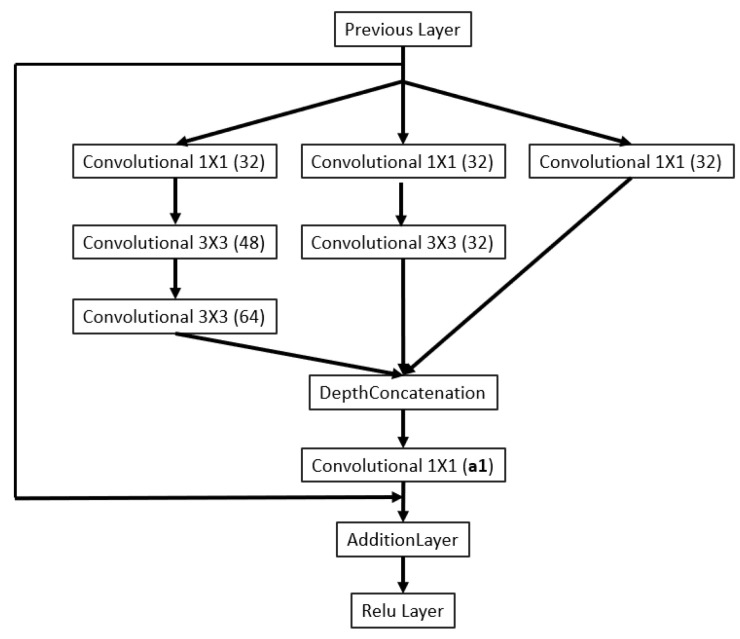
Inception-ResNet-A block.

**Figure 6:**
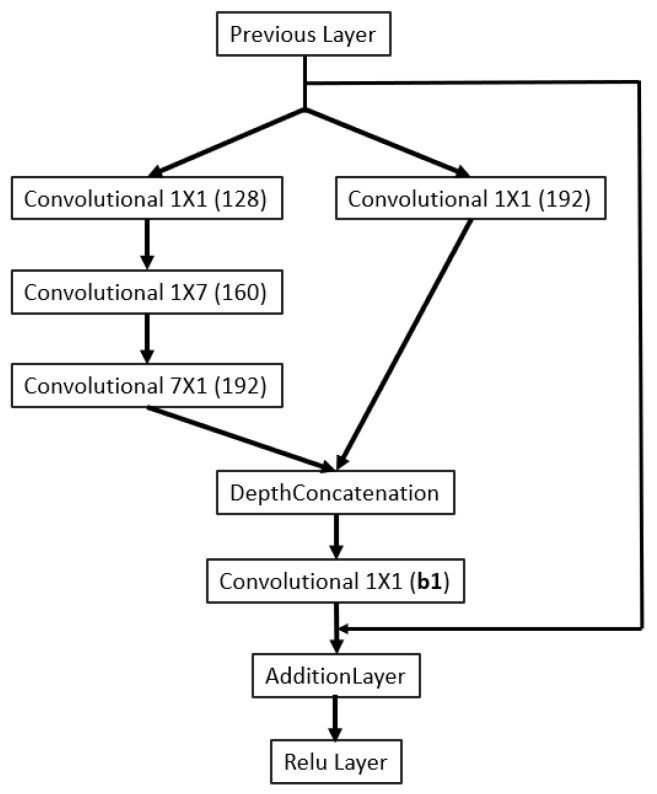
Inception-ResNet-B block.

**Figure 7:**
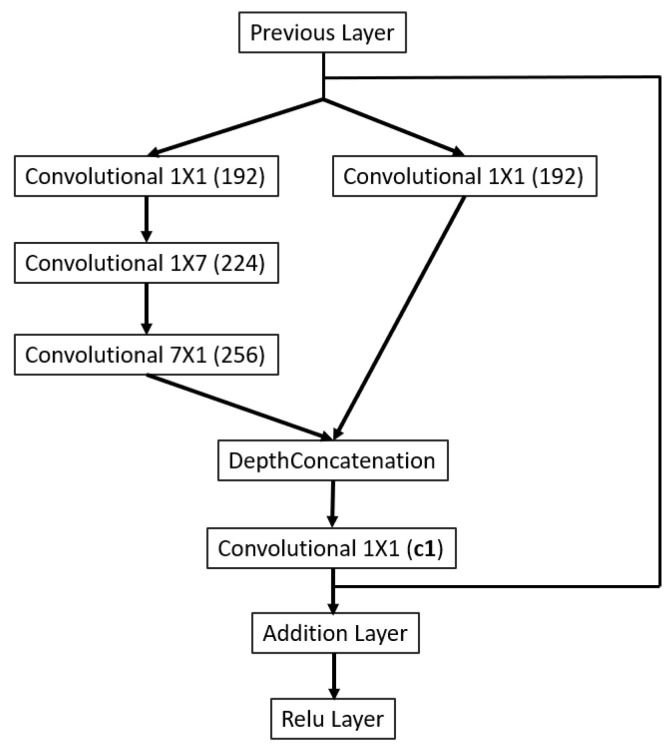
Inception-ResNet-C block.

As illustrated in Figure 2, the backbone of the 3D printing QA network (3D-QAnet) is composed of multiple blocks, including: 1 × Stem block (Figure 3), 1 × Modified Inception-A block (Figure 4), 2 × Modified Inception-ResNet-A blocks (Figure 5), 4 × Modified Inception-ResNet-B blocks (Figure 6) and 2 × Modified Inception-ResNet-C blocks (Figure 7) and YOLO block. Max pooling layers are applied to reduce the spatial dimensions of the input feature map while preserving the important information.

The stem block is the initial part of the proposed network architecture. The block contains a serial of convolution layers to process the input image and extracts low-level features. It was designed to be robust to variations in the input images and to capture a wide range of image features.

The Modified Inception-A block in Figure 4 is one of the basic blocks of the proposed network architecture design to capture features at different scales. It consisted of four parallel branches with different kernel sizes (1 × 1, 3 × 3, 5 × 5) and an average pooling layer that process the input feature map in different ways and concatenate their outputs. The 1 × 1 convolution branch used 1 × 1 convolution to reduce the number of input channels and capture local features. The 3 × 3 average pooling branch used 3 × 3 average pooling to capture global average-pooled features from the input. The 1 × 1 convolutions followed by 3 × 3 convolution branch and 5 × 5 convolution branch captured a combination of local and intermediate-scale features.

The Modified Inception-ResNet-A block in Figure 5 includes residual connections from the ResNet architecture. It was designed to improve gradient flow during training and facilitate faster convergence. The residual connection was added by directly connecting the input feature map to the output of the Inception-A block. This residual connection allows the network to learn both deep and shallow features, helping to mitigate the vanishing gradient problem.

The Modified Inception-ResNet-B block and the Modified Inception-ResNet-C block, as shown in Figure 6 and Figure 7, in the proposed architecture are similar, which included two separate branches (1 × 1 convolution branch and 1 × 1 convolution followed by 7 × 7 convolution branch) for capturing local and intermediate-scale features. The residual structure connects the input feature to the output of the block to keep the gradient flowing.

Three candidate networks, namely 3D-QAnet-160, 3D-QAnet-80, and 3D-QAnet-40, have been proposed in this study based on the schema depicted in Figure 2 and adjustments made to the number of filters in each block. The corresponding number of filters of the corresponding convolution unit is listed in Table 1 for each block.

**Figure 8:**
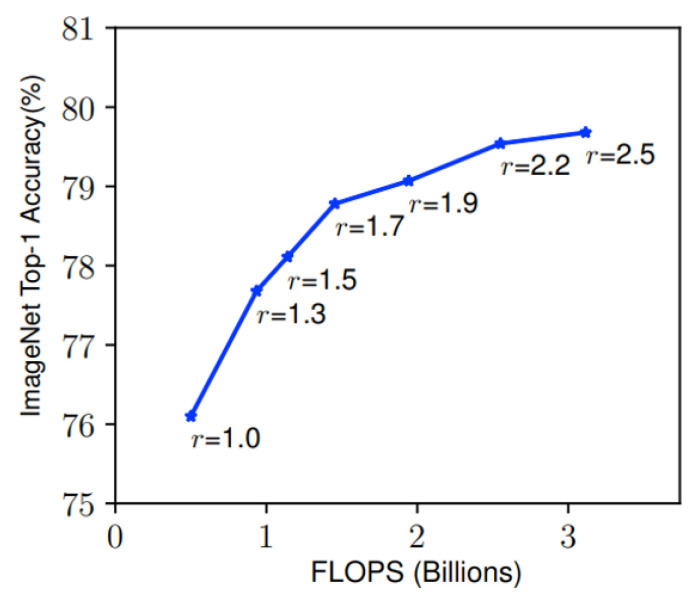
The relationship between the resolution increase and the accuracy (r = 1.0 means the image with resolution 224 × 224, while r = 2.5 means the resolution of 560 × 560) [15].

The proposed neural network in this study employs an enlarged image with higher resolution as input. Previous research has established a correlation between input resolution and accuracy, as depicted in Figure 8 [15]. In the figure, the accuracy improves significantly from r = 1.0 to r = 1.7 and then experiences a gradual increase with further increments in resolution. Therefore, the image with a resolution of 380 × 380 (corresponding to r = 1.7) is chosen as the optimal input resolution for the proposed network.

### 3.2. Creation of Artificial Samples

This study considers three types of failure modes that can occur during 3D printing: indentation, protrusion, and layering. Indentation is a common failure that can result from collisions with debris, scratches, and wear during operation, which can be more severe in the absence of maintenance in space. Protrusion is also considered in this paper as a potential failure mode resulting from outgassing and thermal changes in space during operation. The outgassing and thermal changes in space environment can cause internal forces inside the printed parts and result in deformation on the surface. Layering is a failure caused by several factors, such as inaccurate stepper movement, deformation of the printing beam, and part deformation during the printing process. These factors can impede the smooth movement of the motor and extruder, leading to misalignment of the extruder position in the printing space. Over successive layers, the accumulation of such deviations can result in layering phenomena. Inadequate thermal control during the printing process can exacerbate the occurrence of layering in space due to the lack of convection [14], indicating that the printing system more vulnerable to this failure mode. These failures can significantly affect the quality of the printed parts, leading to major functional failures on a larger scale.

**Figure 9:**
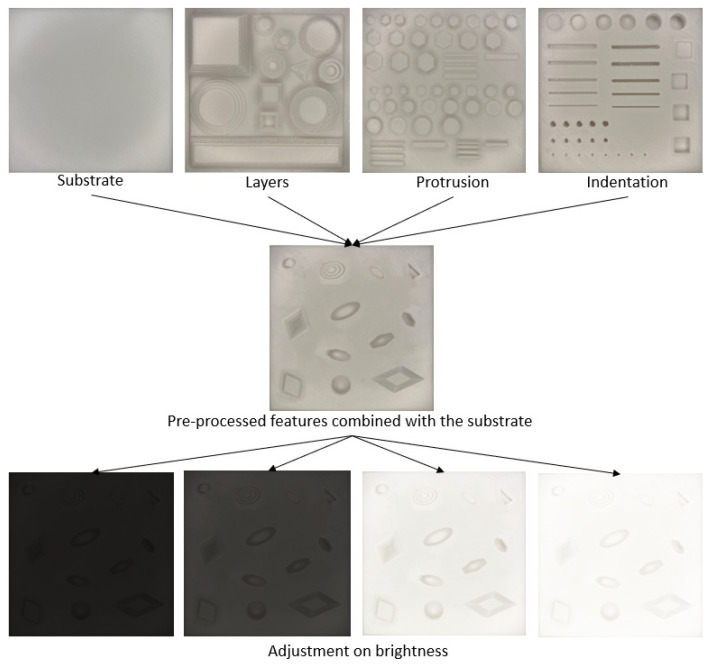
An example of the artificial training sample fabrication process with multiple templates and image processing.

The production of samples with the identified failures can be a time-consuming and material-intensive process that may also damage the printer’s extruder and motors. In this paper, artificial samples of the forementioned failures in various shapes and sizes (as shown in Figure 9) were created and used in the training process. These features were printed on a 100 mm × 100 mm × 5 mm PLA substrate.

For the purpose of training our model, we prepared a diverse set of failure samples with varying shapes and dimensions, which were then printed on a plain substrate. The dents, for instance, were deliberately created with different depths ranging from 1 mm to 4 mm, exhibiting shapes such as triangles, rectangles, rounds, and lines, in order to simulate a wide range of possible damage scenarios. Additionally, the diameters or widths of the dents were varied on the substrate, further adding to the complexity of the failure samples. Similarly, the layering and protrusion failure samples were deliberately designed with diverse shapes and dimensions, including varying the number of layers and the width of each layer on the samples. In this study, the layering samples had a minimum of three layers, enabling our system to effectively detect failures in the early stages of the printing process. High resolution images (3024 × 3024) were then captured of the substrate with the printed samples, and these images underwent multiple pre-processing, including RGB intensity rescaling, rotation, reshaping, and brightness adjustment. This pre-processing process resulted in the generation of over 1000 processed features from the samples. Subsequently, these features were added to 120 plain substrates and the resolution was reduced to 380 × 380 to fit the input of the network. Considering the varied illumination condition in space, the brightness of these samples was adjusted to create the initial training dataset for our network. These carefully curated samples encompassed a variety of, shapes, sizes, and failure modes to simulate the failures in 3D printed parts. The resulting dataset was utilized for both training and testing purposes in our study.

## 4. Results and Discussion

### 4.1. Experiment and Results

**Figure 10:**
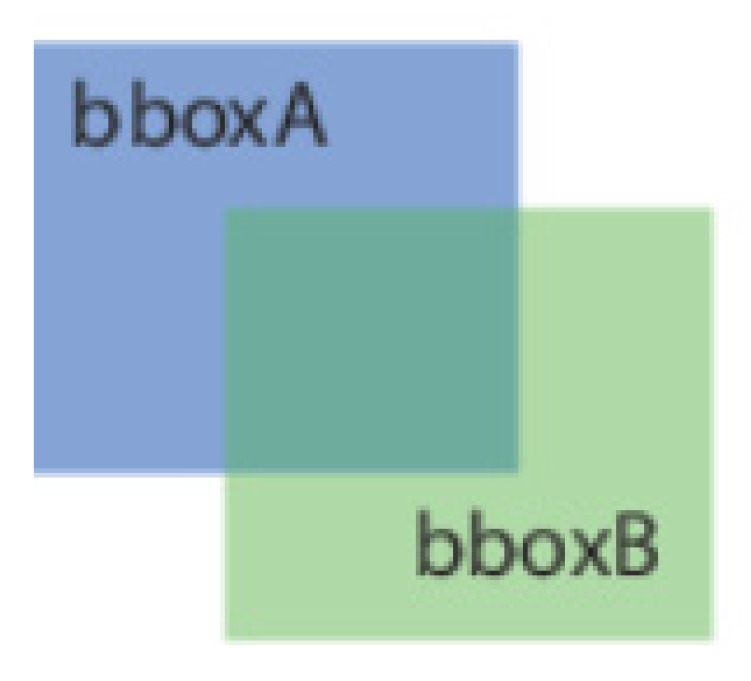
Schematic representation of the Intersection of Union (IOU) [24].

In this study, three proposed networks (3D-QAnet-160, 80, and 40) and four existing networks (GoogleNet, MobileNetV2, and DarkNet19) were utilized as the backbone for the YOLOV3 detection block. These networks were trained with the prepared samples. As the object detection uses bounding box to perform prediction on the targets. The IOU (Intersection over Union), as shown in Figure 10 and Equation (1), was applied to evaluate the precision on the local prediction results. In process, the IOU quantified the extent of overlap between the bounding box of local prediction results and the ground truth bounding box to derive the detection precision. In this paper, the threshold value was set as 0.5, which means if the IOU between the local prediction bounding box and the ground truth bounding box was over 0.5, the result will be regarded as a true positive result.

(1) $$\;IOU\;\left(\mathrm{Intersection}\;\mathrm{over}\;\mathrm{Union}\right)=\frac{Area\;\left(A\cap B\right)}{Area\;\left(A\cup B\right)}$$

Training was carried out using Matlab, and the evaluated losses encompass the localization loss, classification loss, and objectness loss. The box loss encompasses the losses pertaining to the predicted bounding box’s top left corner coordinate, as well as the losses related to the width and height of the bounding box. The object loss reflects the classification failure losses, while the class loss pertains to the confidence losses. The Equations (2)–(4) [25] elucidating the derivation of these losses are presented below:

(2) $$\begin{array}{c} Localization\;Loss=\lambda_{coord}\sum_{i=0}^{S^{2}}\sum_{j=0}^{B}{𝕝}_{ij}^{obj}\left[{\left(x_{i}-{\hat{x}}_{i}\right)}^{2}+{\left(y_{i}-{\hat{y}}_{i}\right)}^{2}\right]+\\ \lambda_{coord}\sum_{i=0}^{S^{2}}\sum_{j=0}^{B}{𝕝}_{ij}^{obj}\left[{\left(\sqrt{w_{i}}-\sqrt{{\hat{w}}_{i}}\right)}^{2}+{\left(\sqrt{h_{i}}-\sqrt{{\hat{h}}_{i}}\right)}^{2}\right] \end{array}$$

(3) $$Classification\;Loss=\sum_{i=0}^{S^{2}}{𝕝}_{i}^{obj}\sum_{cɛclass}^{}{\left(p_{i}\left(c\right)-{\hat{p}}_{i}\left(c\right)\right)}^{2}$$

(4) $$Objectness\;Loss=\sum_{i=0}^{S^{2}}\sum_{j=0}^{B}{𝕝}_{ij}^{obj}{\left((C_{i}-{\hat{C}}_{i}\right)}^{2}+\;\lambda_{noobj}\sum_{i=0}^{S^{2}}\sum_{j=0}^{B}{𝕝}_{ij}^{noobj}{\left((C_{i}-{\hat{C}}_{i}\right)}^{2}]$$

where:

S^2^: number of the grid cells in the target image.

B: number of bounding boxes in each cell.

$x_{i},\;y_{i},\;w_{i},\;h_{i}$: the predicted coordinates and dimensions (width and height) of the bounding box.

${\hat{x}}_{i},\;{\hat{y}}_{i},\;{\hat{w}}_{i},\;{\hat{h}}_{i}$: the ground truth of the bounding box coordinates and dimensions.

$p_{i}\left(c\right)$, ${\hat{p}}_{i}\left(c\right)$: the predicted and ground truth value of the conditional class probability for class c in cell i.

$C_{i},\;{\hat{C}}_{i}$: the predicted and the ground truth confidence value for the bounding box prediction in cell i.

${𝕝}_{ij}^{obj}$: 1 if the jth bounding box in cell i is responsible for the object detected, or 0.

${𝕝}_{i}^{obj}$: 1 if an object is in cell I, or 0.

$\lambda_{coord}$: loss increase from bounding box coordinate predictions.

$\lambda_{noobj}$: loss decrease from confidence predictions the boxes do not have objects.

The tests of the networks were conducted using sample images with various resolutions. The results, as presented in Table 2, reveal the average detectivity (i.e., the average number of successfully detected features in the test images) and the average confidence (i.e., the average value of the confidence of all detected features in test images) of the networks. Under different resolution conditions, it is observed that the 3D-QAnet-40 network can detect the most targets with high confidence. It is also noteworthy that the further increase in image resolution from 380 × 380 to 1600 × 1600 did not significantly affect the detection performance of the networks.

Another important consideration for the proposed QA system is the computation and memory resources required onboard the spacecraft. Given the limited resources available on the manufacturing platform deployed in space, the three proposed networks with relatively better detection performance were compared based on the number of parameters, processing time, and maximum allocated memory (as shown in Table 3), which reflect the computational cost required to run the networks. Less memory cost is important for future application of the network on the platform, while higher processing speed makes the QA assessment process more efficient, particularly when inspecting a large area where multiple images need to be assessed. By contrast, 3D-QAnet-40 requires fewer parameters (2.6 M), less memory (393 MB), and less processing time (350 ms) compared to the other two proposed networks. Considering its good performance in detecting features and its lower computational cost, the 3D-QAnet-40 is a viable option for the QA system of spaceborne 3D printers.

In space, the orientation of the target object will significantly affect the illumination condition. As a result, the brightness on the target surface will vary and the features of the failure will change accordingly. The variable features on the failure can be a challenge for the detection task. In this paper, the network is proposed to be trained with the samples with different brightness. A comparison has been made against the counterpart trained only with normal brightness samples. The average precision and average miss rate (as shown in Equations (5) and (6)) are derived based on testing samples to evaluate the performance. From the results shown in Table 4. It can be seen the network trained with multiple brightness samples shows better performance, particularly on the samples with poor or excessive illumination conditions.

(5) $$Average\;Precision\;\left(AP\right)=\int_{0}^{1}p\left(r\right)dr$$

(6) $$log-Average\;Missrate\;\left(AM\right)=\frac{1}{n}\;\sum_{i=1}^{n}a_{i}$$

where: *p*(*r*) is the function between the precision (*p*) and recall (*r*).

a_i_ represents the corresponding miss rate value at nine evenly spaced False Positive Per Image (FPPI) rates between 10^−2^ and 10^0^.

**Figure 11:**
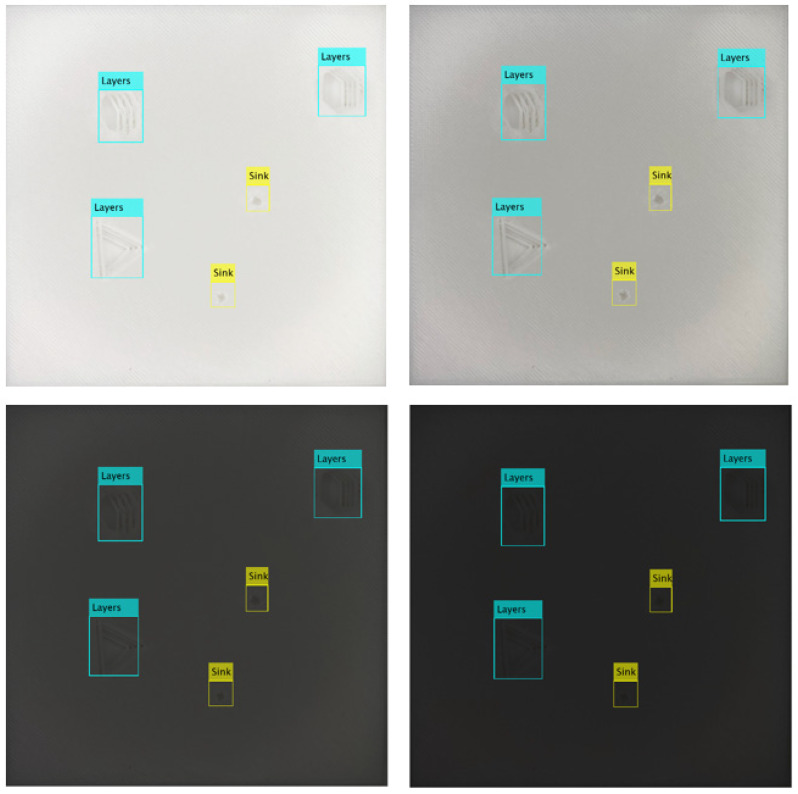
The detection under different brightness with actual failures from failed 3D printed part.

Some of the testing results are shown in Figure 11 based on the failure features collected from failed printed pieces. The trained network can perform well to detect all the failures in varying brightness conditions.

### 4.2. Discussion

Theoretically, higher resolution input to the neural network should enhance the extraction of detailed feature maps, thereby improving the accuracy of detection results. However, in this study, a clear improvement in results with increasing input resolution was not observed. This may be due to the fact that the network was trained on images with a resolution of 380 × 380, which limits its ability to capture finer details that could be obtained from higher resolution images during training. Consequently, the high resolution features with greater details may not significantly contribute to improving accuracy to a large extent. However, considering the proposed application scenario, handling higher resolution input images may incur higher computational and storage costs. In addition, based on the findings related to accuracy, computation cost, and input resolution, the input image with a resolution of 380 × 380 achieved a good balance between these factors.

The impact of illumination changes on input images can significantly affect detection results, as critical features may undergo variations. Therefore, training the network with samples of varying brightness levels can lead to improved accuracy and reduced miss rates. Particularly, when detecting failures under excessive illumination, the network trained with single brightness samples may struggle to detect layering and protrusion failures effectively, whereas its counterparts trained with varied brightness samples still exhibit better performance, achieving accuracies of 0.73 and 0.74, respectively. Even under normal brightness conditions, the network trained with varying brightness samples consistently demonstrates better performance. This provides evidence that illumination variation is an important factor to be considered when designing a failure detection system.

**Figure 12:**
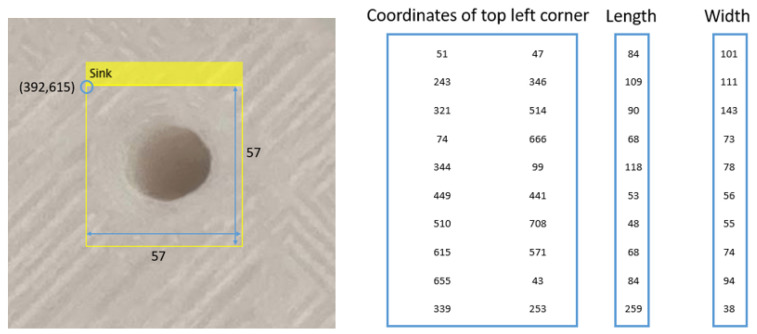
An example of the data obtained from the QA system (failure category, coordinate of the top left corner, length, and width of the failure target area). If multiple failures detected, the data will be stored in a matrix format.

Furthermore, the detection results obtained from the proposed network can provide location and dimension information, which can be utilized to support the repair process of damaged or failed 3D printed parts. Specifically, the QA system equipped with the detection network can identify and locate each failure using a rectangular box. The detection result data will include the coordinate information at the top left corner of the box, as well as the length and width of the box, as demonstrated in Figure 12. Based on the data acquired, a point cloud scanner on the manufacturing unit can scan the areas of interest with failures to gather detailed information and guide extruder on the 3D printer to repair the failures [26]. In addition to the quantity and category of the identified failures, the location and dimension information can provide further details to enhance the judgment process of the QA system.

The 3D-QAnet-based QA system uses the detection results, which provide information on the quantity, category, location, and dimension of the detected failures, to determine whether the inspected area meets the pass/fail criteria. These criteria are adaptable and are contingent on the function and application context of the targeted part.

The implementation of the repairing function, supported by the QA data, can effectively reduce material waste resulting from printing and operation. Given the high transportation cost for resupply in space, repairing damaged 3D printed parts can further lower the cost of space missions. The location and dimension information of failures captured by the QA system plays a crucial role in enabling the space-based 3D printer to perform the repairing process. With the information from the QA system, the 3D camera on the platform can scan the target areas highlighted by the rectangular boxes and generate 3D images of the failures. The printing data obtained from these images can then be used to guide the printer to complete the repairing process.

## 5. Conclusions

This paper presents a QA system based on computer vision and CNN detection network for on-orbit 3D printing. The objective of the system is to reduce material waste and lower the cost of resupplying materials in space by enabling an automatic closed control loop for the printing process with the QA system. The proposed system was developed using computer vision-based CNNs detection networks. The proposed networks (3D-QAnet-40, 3D-QAnet-80, and 3D-QAnet-160) along with another four existing networks (GoogleNet, MobileNetV2 and DarkNet19) used as the backbone of YOLO were studied in this paper. The detection performance of the networks is evaluated with sample images at varying resolutions and brightness, and 3D-QAnet-40 was found to perform the best. When selecting the networks to be used on the space-based manufacturing platform, the processing time and memory demand are important factors to consider, given the limited onboard computation resources. Among the discussed networks, 3D-QAnet-40 was found to present the best detection performance with less process time (350ms) and memory cost (393MB). The system’s reliability in space environment is further tested with samples under different illumination conditions. Additionally, the system’s data can be used to fix damaged 3D printed pieces by providing location and dimension information. By integrating the 3D camera onto the manufacturing platform, the feature information of failures can be extracted and used to plan the repairing route of the extruder.

Despite the promising results presented in this study, there are still areas for further research and development to fully implement the proposed QA system for space-based 3D printing. One area that requires attention is the testing of the system on an embedded onboard control unit. Additionally, the design of robotic arms and the integration of camera(s) and will be necessary to obtain high-quality 3D images of the printed pieces.

## Figures and Tables

**Table 1:** The number of filters of each block applied in the three variants presented in this paper (the changes on the variable values represent different number of filters in the corresponding block in each variant).

Variables	Blocks	3D-QAnet-40	3D-QAnet-80	3D-QAnet-160
a	Modified Inception-A block (Figure 4)	12	24	48
b	Modified Inception-A block (Figure 4)	8	16	32
c	Modified Inception-A block (Figure 4)	6	12	24
d	Modified Inception-A block (Figure 4)	8	16	32
e	Modified Inception-A block (Figure 4)	8	16	32
f	Modified Inception-A block (Figure 4)	12	24	48
g	Modified Inception-A block (Figure 4)	12	24	48
a1	Inception-ResNet-A blocks (Figure 5)	40	80	160
b1	Inception-ResNet-B blocks (Figure 6)	40	80	160
c1	Inception-ResNet-C blocks (Figure 7)	40	80	160

**Table 2:** The experiment on images with multiple resolutions.

Resolution	Items	3D-QAnet-160	3D-QAnet-80	3D-QAnet-40	MobileNet	GoogleNet	DarkNet19
380 × 380	Detectivity	0.764	0.709	0.791	0.609	0.645	0.564
	Confidence	0.892	0.907	0.904	0.856	0.875	0.864
600 × 600	Detectivity	0.727	0.709	0.827	0.591	0.655	0.573
	Confidence	0.917	0.902	0.893	0.858	0.864	0.861
800 × 800	Detectivity	0.764	0.709	0.809	0.582	0.655	0.564
	Confidence	0.897	0.902	0.905	0.860	0.869	0.856
1600 × 1600	Detectivity	0.745	0.700	0.827	0.591	0.645	0.564
	Confidence	0.896	0.892	0.916	0.845	0.867	0.869

**Table 3:** The performance results of the networks for image detection tasks (the test is performed based on the same testing images).

	Parameters (M)	Process Time (ms)	Maximum Allocated Memory (MB)
3D-QAnet-160	3.45	557	399
3D-QAnet-80	2.87	372	421
3D-QAnet-40	2.6	350	393

**Table 4:** The comparison between the networks trained with varying and single brightness samples.

Sample	Failure	Trained with Varying Brightness Samples		Trained with Single Brightness Samples	
		Average Precision	Average MissRate	Average Precision	Average MissRate
Normal Sample	Dents	0.876	0.199	0.817	0.244
	Protrusion	0.703	0.449	0.723	0.405
	Layering	0.750	0.250	0.648	0.485
Exposure Sample	Dents	0.900	0.133	0.638	0.449
	Protrusion	0.734	0.503	0.000	1.000
	Layering	0.714	0.286	0.107	0.893
Dark Sample	Dents	0.898	0.156	0.857	0.219
	Protrusion	0.766	0.439	0.741	0.449
	Layering	0.747	0.280	0.635	0.410

## Data Availability

Available on request.

## References

[1] Shifa M., Tariq F., Khan F., Toor Z.S., Baloch R.A. (2020). Towards Light Weight Multifunctional Hybrid Composite Housing for Satellite Electronics Towards Light Weight Multifunctional Hybrid Composite Housing for Satellite Electronics. Mater. Res. Express.

[2] Leach, Neil (2014). 3D printing in space. Archit. Des..

[3] Gaskill M. (2019). Solving the Challenges of Long Duration Space Flight with 3D Printing. https://www.nasa.gov/mission_pages/station/research/news/3d-printing-in-space-long-duration-spaceflight-applications/.

[4] Reitz B., Lotz C., Gerdes N., Linke S., Olsen E., Pflieger K., Sohrt S., Ernst M., Taschner P., Neumann J. (2021). Additive Manufacturing Under Lunar Gravity and Microgravity. Microgravity Sci. Technol..

[5] Owens A., De Weck O., Stromgren C., Goodliff K.E., Cirillo W. Supportability challenges, metrics, and key decisions for future human spaceflight. Proceedings of the AIAA SPACE and Astronautics Forum and Exposition.

[6] Owens A., De Weck O. Systems Analysis of In-Space Manufacturing Applications for the International Space Station and the Evolvable Mars Campaign. Proceedings of the AIAA SPACE 2016.

[7] Loff S. (2021). International Space Station’s 3-D Printer. https://www.nasa.gov/content/international-space-station-s-3-d-printer.

[8] Prater T., Werkheiser N., Ledbetter F., Timucin D., Wheeler K., Snyder M. (2019). 3D Printing in Zero G Technology Demonstration Mission: Complete Experimental Results and Summary of Related Material Modeling Efforts. Int. J. Adv. Manuf. Technol..

[9] Papadopoulos L. (2021). ESA to Test 3D Printing in Space Using Scrap Metals from the Moon. https://interestingengineering.com/esa-to-test-3d-printing-in-space-using-scrap-metals-from-the-moon.

[10] Kubi.S 2020. https://3dprintingindustry.com/news/china-celebrates-its-first-set-of-3d-printing-tests-in-space.

[11] (2019). NASA. https://spinoff.nasa.gov/Spinoff2019/ip_2.html.

[12] Ngo T.D., Kashani A., Imbalzano G., Nguyen K.T., Hui D. (2018). Additive Manufacturing (3D Printing): A Review of Materials, Methods, Applications and Challenges. Compos. Part B Eng..

[13] Bengio Y., LeCun Y. (2007). Scaling learning algorithms towards AI. Large-Scale Kernel Mach..

[14] Szegedy C., Vanhoucke V., Ioffe S., Shlens J., Wojna Z. Rethinking the Inception Architecture for Computer Vision. Proceedings of the IEEE Conference on Computer Vision and Pattern Recognition.

[15] Ge Z., Liu S., Wang F., Li Z., Sun J. (2021). YOLOX: Exceeding YOLO Series in 2021. arXiv.

[16] Nepal U., Eslamiat H. (2022). Comparing YOLOv3, YOLOv4 and YOLOv5 for Autonomous Landing Spot Detection in Faulty UAVs. Sensors.

[17] Xie Z., Sun T., Kwan T., Wu X.X. (2020). Motion Control of a Space Manipulator Using Fuzzy Sliding Mode Control with Reinforcement Learning. Acta Astronaut..

[18] Tang J., Hocksun T., Wu X. (2022). Extrusion and thermal control design of an on-orbit 3D printing platform. Adv. Space Res..

[19] Tan M., Le Q. Efficientnet: Rethinking model scaling for convolutional neural networks. Proceedings of the International Conference on Machine Learning.

[20] Huang Y., Cheng Y., Bapna A., Firat O., Chen D., Chen M., Lee H., Ngiam J., Le Q.V., Wu Y. (2019). Gpipe: Efficient training of giant neural networks using pipeline parallelism. Adv. Neural Inf. Process. Syst..

[21] Szegedy C., Ioffe S., Vanhoucke V., Alemi A. Inception-v4, inception-resnet and the impact of residual connections on learning. Proceedings of the AAAI Conference on Artificial Intelligence.

[22] Szegedy C., Liu W., Jia Y., Sermanet P., Reed S., Anguelov D., Erhan D., Vanhoucke V., Rabinovich A. Going Deeper with Convolutions. Proceedings of the IEEE Conference on Computer Vision and Pattern Recognition.

[23] He K., Zhang X., Ren S., Sun J. Deep residual learning for image recognition. Proceedings of the IEEE Conference on Computer Vision and Pattern Recognition.

[24] Matlab. https://www.mathworks.com/help/vision/.

[25] Redmon J., Divvala S., Girshick R., Farhadi A. You only look once: Unified, real-time object detection. Proceedings of the IEEE Conference on Computer Vision and Pattern Recognition.

[26] Tang J., Wu X. (2023). Design of Quality Assessment System for On-Orbit 3D Printing Based on 3D Reconstruction Technology. Int. J. Ind. Manuf. Eng..

